# Cervical Cancer Prognosis and Diagnosis Using Electrical Impedance Spectroscopy

**DOI:** 10.2478/joeb-2021-0018

**Published:** 2021-12-27

**Authors:** Ping Li, Peter E. Highfield, Zi-Qiang Lang, Darren Kell

**Affiliations:** 1 Department of Automatic Control and Systems Engineering, The University of Sheffield, Sheffield, UK; 2 Zilico Ltd, Manchester, UK

**Keywords:** Electrical impedance spectroscopy (EIS), cervical cancer, diagnosis, prognosis, Cole model, spectrum curve fitting, logistic regression, classification

## Abstract

Electrical impedance spectroscopy (EIS) has been used as an adjunct to colposcopy for cervical cancer diagnosis for many years, Currently, the template match method is employed for EIS measurements analysis, where the measured EIS spectra are compared with the templates generated from three-dimensional finite element (FE) models of cancerous and non-cancerous cervical tissue, and the matches between the measured EIS spectra and the templates are then used to derive a score that indicates the association strength of the measured EIS to the High-Grade Cervical Intraepithelial Neoplasia (HG CIN). These FE models can be viewed as the computational versions of the associated physical tissue models. In this paper, the problem is revisited with an objective to develop a new method for EIS data analysis that might reveal the relationship between the change in the tissue structure due to disease and the change in the measured spectrum. This could provide us with important information to understand the histopathological mechanism that underpins the EIS-based HG CIN diagnostic decision making and the prognostic value of EIS for cervical cancer diagnosis. A further objective is to develop an alternative EIS data processing method for HG CIN detection that does not rely on physical models of tissues so as to facilitate extending the EIS technique to new medical diagnostic applications where the template spectra are not available.

An EIS data-driven method was developed in this paper to achieve the above objectives, where the EIS data analysis for cervical cancer diagnosis and prognosis were formulated as the classification problems and a Cole model-based spectrum curve fitting approach was proposed to extract features from EIS readings for classification. Machine learning techniques were then used to build classification models with the selected features for cervical cancer diagnosis and evaluation of the prognostic value of the measured EIS. The interpretable classification models were developed with real EIS data sets, which enable us to associate the changes in the observed EIS and the risk of being HG CIN or developing HG CIN with the changes in tissue structure due to disease. The developed classification models were used for HG CIN detection and evaluation of the prognostic value of EIS and the results demonstrated the effectiveness of the developed method. The method developed is of long-term benefit for EIS–based cervical cancer diagnosis and, in conjunction with standard colposcopy, there is the potential for the developed method to provide a more effective and efficient patient management strategy for clinic practice.

## Introduction

**Fig.1:**
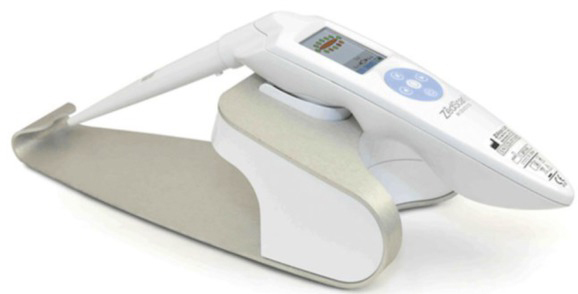
The ZedScan handset for making the EIS measurements used in this paper. The handset is shown placed on the base.

Cervical cancer is the third most common cancer for women in the world [1]. Screening for cervical cancer is usually performed using a multi-tiered paradigm which begins with the Papanicolaou (Pap) smear with human papillomavirus (HPV) co-testing, followed by colposcopy guided biopsy and prevention of cervical cancer depends on colposcopic detection and treatment of high-grade cervical intraepithelial neoplasia (HG-CIN) in women referred with abnormal cytology. Cervical epithelium is a highly structured and stratified tissue that exhibits changes as it progresses from normal epithelium to HG-CIN. These changes are associated with losses in the layer of flattened epithelial cells close to the surface of the cervix, and increases in both the nuclear/cytoplasmic ratio and the extracellular space. All of these changes caused by the disease will eventually lead to a change in the impedance compared with a normal cervix. As a result, in contrast to colposcopy, the Electrical Impedance Spectroscopy (EIS) can be used as a non-visual technique to image epithelia. The research on HG-CIN detection using EIS had been carried out for many years [24] and the EIS measurement device ZedScan^TM^ (see Fig.1) has been developed for real-time diagnostics [5].

Currently, EIS has been used as an adjunct to colposcopy for HG-CIN detection to improve the diagnostic performance [6]. The impedances are measured with ZedScanTM at 14 frequencies, logarithmically spaced between 76 Hz and 625 kHz. The template matching method has been used for analysing the 14-frequency EIS spectra measured from a maximum of 12 reading sites around the cervix for diagnosis [5,6] where the measured spectra are compared with the ‘template’ spectra generated from the 3-D finite element models of the normal and abnormal cervical tissues and matching between the measured spectra and the templates is made using the least squares method, finally the results from matching are then used to generate a probability index for the detection of HG-CIN.

Complementary to colposcopy, EIS has been shown able to differentiate between normal, pre-cancerous and cancerous tissues. It plays an important role in improving performance of colposcopy-only diagnosis as shown in previous studies [5,6]. The template matching method used in the previous studies for EIS data analysis relies on the template spectra generated from the 3-D finite element models of the normal and abnormal cervical tissues. These 3-D finite element models can be viewed as the computational versions of the associated physical tissue models. Building quality 3-D finite element model to obtain template spectra is a time and effort demanding job, requiring extensive domain knowledge and involving detailed histopathological analysis of normal and diseased tissues, and in some cases this may be difficult. This hinders the extension of EIS-based technique in new areas of medical diagnosis where template spectra are not available. In addition to producing a probability index used for HG CIN detection, it would also be desirable to be able to establish a direct link between this probability index and the associated tissue structure properties as this will provide important information for us to understand the histopathological mechanism that underpins the EIS-based cervical cancer prognosis and diagnosis, and to improve interpretability of the diagnostic results.

The problems mentioned above are addressed in this paper. A EIS data-driven modelling based approach was developed for EIS measurement data analysis. The new approach does not rely on the template spectra and HG CIN detection was formulated as a classification problem where a Cole model-based spectrum curve fitting method was proposed to extract the features from EIS readings and a logistic regression model was employed for performing the classification which revealed the association between the tissue structure changes caused by disease and the changes in the measured EIS through the Cole parameter estimates. The developed approach was also used for a longitudinal EIS data analysis to evaluate prognostic value of the EIS for cervical cancer diagnosis and the results are reported in this paper.

## Methods

The EIS measurements with confirmed diagnostic outcomes used in the study presented in this paper were taken from 1704 women and there were at least 8 impedance spectra (taken from different reading sites around the cervix) for each individual. For HG CIN detection, the entire population was divided into two groups, those women with confirmed HG CIN which had N=528 ( 30.99% ) and those women without confirmed HG CIN which had N=1176, The objective of the study is to develop a template-free method for separating these two groups using the measured EIS. Among 1176 women with non-HG CIN, 569 women were followed up to three years after their initial colposcopy. Of these, 35 (6.15%) women were found to develop HG-CIN within three follow-up years and 534 women were not. The EIS data of these 569 women were used for a longitudinal study to evaluate prognostic value of the EIS for cervical cancer diagnosis. In this case, the entire population of size 569 was divided into two groups, with one group including all women who had developed HG-CIN within three follow-up years and another group including women who had not. The objective of this longitudinal study is to see if it is possible to identify women who are likely to develop HG-CIN within three follow-up years based on the EIS measurement taken at their initial colposcopy so as to evaluate the prognostic value of EIS for cervical cancer diagnosis.

The basic idea behind the EIS-based template match method for HG-CIN detection as mentioned above is to identify the difference in spectrum shapes between diseased and non-diseased tissues by directly comparing the measured spectra with the template spectra to generate features for diagnosis. In contrast to direct comparison, a model-based spectrum curve fitting approach was proposed in this paper to extract features from EIS readings for diagnosis with an aim to reveal how the tissue structure changes due to disease might be reflected in the measured

EIS, in addition to detecting HG CIN. Specifically, we try to fit a model to the measured spectrum, and then derive the required features for disease detection from the fitted model parameters.

This study was a service evaluation carried out in the Jessop Wing Colposcopy clinic in Sheffield and so no ethical approval was required [6]. All patient data mentioned above was anonymised.

### Model-based bio-impedance spectrum curve fitting

The commonly used model for biological tissue impedance is the Cole equation of the following form [7,2]:


(1) $$Z(f)=R_{\infty }+\frac{R_{0}-R_{\infty }}{1+{\left(\frac{jf}{f_{c}}\right)}^{1-\alpha }}$$

This is an equivalent model that is commonly used by researchers in the field to describe the relationship between the measured tissue impedance *Z* and frequency *f*, In equation (1), $R_{0}\text{ and }R_{\infty }$are the resistances at zero and infinite frequency that will determine the values of impedance spectrum at low and high frequency bands respectively. *f* is the frequency of excitation current used in measurement and 14 logarithmically spaced frequencies (with $f_{1}=76\;\text{ Hz and }f_{14}=625\text{ kHz}$are used in measurement. $f_{c}$is often referred to as the characteristic frequency and αis a constant (0≤α≤1).These four model parameters are associated with the tissue structure and properties under investigation and need to be determined from the measured EIS data.

**Fig.2:**
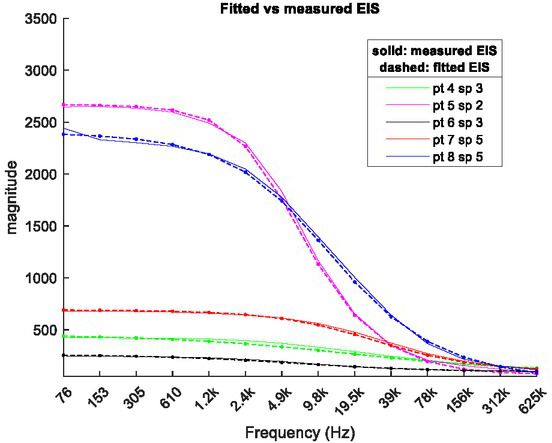
Comparison between measured and model fitted EIS

Equation (1) is a nonlinear complex model and spectrum curve fitting for determination of the model parameters can be formulated as a complex nonlinear optimization problem. This can be solved using the trust-region-reflective algorithm [8], subject to the bounds determined with the measured EIS spectra. Fig. 2 below shows some typical results of Cole model-based EIS fitting with the aforementioned algorithm, where solid lines represent the measured spectra and dashed lines represent the model fitted spectra.

## Feature extraction from fitted model

The structure of biological tissue is complex and the impedance change with frequency will depend upon many factors, such as cellular arrangement (layering of cells), extracellular space, cell size, conductivity of extracellular fluid, thickness of cell membrane, electrical properties of cell membrane and so on. When cervical epithelium progresses from normal epithelium to high-grade CIN, the tissue properties mentioned above will also be altered which are reflected in the changes in the measured EIS spectra, hence allowing EIS to be used for disease detection [9]. Ultimately, these changes will lead to changes in the four estimated parameters $R_{0},R_{\infty },f_{c}\text{ and }\alpha$of the Cole model (1), this enables us to derive features from four estimated Cole model parameters for HG CIN detection using classification techniques.

A commonly used interpretation [10] of the four Cole model parameters for tissue structure is that the inverse of extracellular volume determines $R_{0},$the inverse of the total volume determines $R_{\infty }$, cell sizes determine $f_{c},$which is the centre of the dispersion, and α is determined by the inhomogeneity of the cells within the dispersion. The conductivity of the intracellular and extracellular spaces will also affect both $R_{0}\text{ and }R_{\infty }$, If the classification model for HG CIN detection can be constructed with the features derived from the four Cole parameter estimates, the above interpretation will provide us with valuable information to understand how the tissue structure changes due to disease might be reflected in the observed EIS spectrum. This would, in turn, be very helpful for us to understand the fundamental mechanism that underpins the EIS based HG CIN detection.

When used as an adjunct to colposcopy, EIS spectral measurements are made at up to 12 reading sites around cervix (minimum number of sites is 8) of individual women. As the lesion can either be large to cover many sites or be small covering a few sites, or even a single site on cervix, two types of feature were derived from Cole model parameter estimates. The first type of feature consists of the four Cole model parameter estimates (denoted as: ${\bar{R}}_{\infty },{\bar{R}}_{0},{\bar{f}}_{c},\bar{\alpha })$of the mean spectrum over all reading sites of an individual woman which aims to provide information for detecting large lesions that cover many reading sites. The second type of feature consists of the four maximum differences of Cole model parameter estimates over all (up to 12) reading sites around the cervix of an individual woman defined as follows:


(2) $$\begin{array}{l} \Delta R_{\infty }=\underset{i}{\max}R_{\infty }^{i}-\underset{i}{\min}R_{\infty }^{i}\\ \Delta R_{0}=\underset{i}{\max}R_{0}^{i}-\underset{i}{\min}R_{0}^{i}\\ \Delta f_{c}=\underset{i}{\max}f_{c}^{i}-\underset{i}{\min}f_{c}^{i}\;\left(i=1,2,\cdots ,12\right)\\ \Delta \alpha =\underset{i}{\max}\alpha^{ı}-\underset{i}{\min}\alpha^{ı} \end{array}$$

The features defined by (2) can be viewed as a measure of spatial inhomogeneity of the tissue around cervix and are expected to provide information for detecting small lesions presented in a few or just a single reading site. The rationale behind this is that, if there are no lesions around cervix, EIS taken at all sites will have approximately the same shape, thus similar Cole model parameter estimates are expected when performing spectrum curve fitting and the differences defined by equation (2) will be small. However, if a lesion does exist and only presents in a few or a single site, the EIS taken at these sites will significantly differ from those taken at sites where no lesions were present. Hence, the differences defined in equation (2) will be large. To sum up, using both Cole model parameter estimates associated with the mean spectrum and the differences defined by equation (2) (i.e. ${\bar{R}}_{\infty },{\bar{R}}_{0},{\bar{f}}_{c},\bar{\alpha },\Delta R_{\infty },\Delta R_{0},\Delta f_{c},\Delta \alpha )$from individual women as features will allow both large and small lesions to be detected.

### Feature selection using multivariate analysis of variance

Cervical cancer diagnosis, or more specifically, HG CIN detection using EIS can be viewed as a problem of detecting changes in the measured EIS taken around cervix which are caused by the change in tissue structure due to HG CIN. Whereas, the evaluation of prognostic value of EIS for cervical cancer diagnosis can be viewed as a problem of detecting early signs in the EIS taken at the initial colposcopy which is caused by the incipient change in tissue structure as neoplasia develops (i.e. the early stage in the evolution of neoplasia). Both problems are formulated as a classification problem in this paper and machine learning technique (see e.g. [11, 12]) was employed to solve the problem. Specifically, the feature/predictor vector defined as:


(3) $$x={\left[{\bar{R}}_{\infty },{\bar{R}}_{0},{\bar{f}}_{c},\bar{\alpha },\Delta R_{\infty },\Delta R_{0},\Delta f_{c},\Delta \alpha \right]}^{T}$$

derived from the fitted Cole model in last subsection will be used to build a predictive model for solving this classification problem.

The complexity of any predictive model for classification depends on the number of input dimensions (i.e. the number of features to be used). In the last subsection, eight handcrafted features defined in (3) have been derived from the EIS measurements. However, the effect of neoplasia on the four Cole model parameters, hence the features derived, is complex and some of these features may be redundant or not informative. Statistically, it is often more attractive to estimate a simpler model with non-informative features being removed as this usually leads to a reduced estimation variance and improved robustness in prediction, and also prevents over fitting for the given data set of fixed size. From a practical point of view, a simpler model may also be more interpretable. Our early study [13] had shown that using any single feature collected in x was not statistically sufficient to allow a separation of two groups. To this end, multivariate analysis of variance (MANOVA) [14] was used for evaluating and ranking the capability of the various combinations of the derived features collected in ***x*** to separate two groups for both problems of HG CIN detection and prognostic value evaluation. The results from analysis are summarized in Table 1 and 2, which enable us to identify the most informative feature combinations to be use for building classification models for HG CIN detection and evaluation of prognostic value of EIS respectively.

**Table.1:** *p*-values from MANOVA using EIS data taken from 1704 women for HG CIN detection

Feature combinations	*p*-values	Feature combinations	*p*-values
${\bar{R}}_{0},\bar{\alpha },\Delta R_{0}$	1.1003 × 10^−31^	${\bar{R}}_{0},\bar{\alpha },\Delta R_{0},\Delta f_{c}$	5.0124 × 10^−31^
${\bar{R}}_{0},\Delta R_{0}$	1.5861 × 10^−31^	${\bar{R}}_{\infty },{\bar{R}}_{0},\Delta R_{\infty },\Delta R_{0}$	5.3276 × 10^−31^
${\bar{R}}_{0},\bar{\alpha },\Delta R_{\infty },\Delta R_{0}$	2.6287 × 10^−31^	${\bar{R}}_{0},\Delta R_{0},\Delta f_{c}$	6.5955 × 10^−31^
${\bar{R}}_{0},\Delta R_{\infty },\Delta R_{0}$	3.1665 × 10^−31^	${\bar{R}}_{0},\Delta R_{0},\Delta \alpha$	7.2683 × 10^−31^
${\bar{R}}_{0},\bar{\alpha },\Delta R_{0},\Delta \alpha$	3.4687 × 10^−31^	${\bar{R}}_{0},{\bar{f}}_{c},\bar{\alpha },\Delta R_{0}$	7.4318 × 10^−31^

**Table.2:** *p*-values from MANOVA using EIS data taken at initial colposcopy of 569 women for evaluation of prognostic value of EIS

Feature combinations	*p*-values	Feature combinations	*p*-values
$\bar{\alpha },\Delta \alpha$	0.0168	${\bar{f}}_{c},\Delta \alpha$	0.0286
$\bar{\alpha },\Delta R_{0}$	0.0231	${\bar{R}}_{0},\bar{\alpha }$	0.0295
${\bar{f}}_{c},\bar{\alpha }$	0.0256	${\bar{R}}_{\infty },\bar{\alpha }$	0.0296
$\bar{\alpha },\Delta R_{\infty }$	0.0274	${\bar{f}}_{c},\bar{\alpha },\Delta \alpha$	0.0314
$\bar{\alpha },\Delta f_{c}$	0.0275	${\bar{R}}_{0},\bar{\alpha },\Delta \alpha$	0.0335

Table 1 shows the results for comparing the multivariate means of the different combination of features from the two groups of women (i.e. HG CIN *vs* no HG CIN) with entire size of 1704 for HG CIN detection. Columns 1 and 3 in Table 1 specify the feature combinations to be compared and columns 2 and 4 show the corresponding *p*-values for testing whether the specified combinations have the same means (i.e. the corresponding mean vectors lie in a space of dimension 0). The smaller the *p*-value, the more confidence there is that the two groups have different means when compared using a particular feature combination. Hence, these *p*-values can be used as the indices to measure the capability of the corresponding feature combinations to separate two groups. Extensive multivariate analysis of variance had been carried out and Table 1 lists the ten feature combinations with the smallest *p*-values among all possible combinations of eight features. From Table 1 and for the given EIS data set of size 1704, the *p*-values are very small hence we can safely reject the null hypothesis that the means of two groups are the same. Table 1 also shows that using more features does not necessarily increase capability to separate two groups. This indicates that some features in x may be redundant and the most informative features for separating two groups (HG CIN *vs* non-HG CIN) are associated with the extracellular volume and inhomogeneity of the cells within the tissue $({\bar{R}}_{0}\text{and}\bar{\alpha })$as well as the spatial inhomogeneity of the tissue around cervix $(i.e.\;\Delta R_{0}).$These results provide useful information for selecting features to build classifier for HG CIN detection.

Similarly, Table 2 below shows the results for comparing the multivariate means of the different combinations of features from the two groups of women (i.e. HG CIN developed *vs* no HG CIN developed within three follow-up years) with entire size of 569 for evaluating the prognostic value of EIS for cervical cancer diagnosis. Ten different feature combinations with the smallest *p*-values among all possible combinations of eight features are listed in Table 2.

It can be seen that, in comparison with Table 1, the *p*-values shown in Table 2 are much larger than those in Table 1, This suggests that there is a lower level of confidence (in comparison with the case of HG CIN detection using EIS) in the ability to separate the two groups using these feature combinations. This might be expected, because it will be more difficult to detect early signs of neoplasia in the measured EIS than to detect the more substantial changes caused by severe neoplasia or HG CIN. Nonetheless, the results do reach statistical significance (at the usual 5% significance level) to allow a rejection of null hypothesis that the means of two groups are the same. Again, Table 2 also shows that using more features does not necessarily increase the capability to separate two groups. But in this case, the most informative features for separating two groups are associated with the inhomogeneity of the cells within the tissue and the spatial inhomogeneity of tissue around cervix $(i.e.\;\bar{\alpha }\text{ and }\Delta \alpha ).$From a histopathological perspective, this is reasonable. Inhomogeneity of the cells (i.e. cell diversity) within the tissue and the spatial inhomogeneity of tissue around cervix are the properties associated with the evolution of neoplasia, hence are features for detecting early sign in the measured EIS. Once the neoplasia becomes more severe and/or has transferred into HG CIN, in addition to cellular diversity, another property i.e. extracellular volume that determines ${\bar{R}}_{0}$becomes the main property to differentiate between normal and cancerous tissues as previously described.

### Stratified cross-validation for classification model determination

As discussed above, the problems to be solved in this study can be viewed as a binary classification problem, once the features to be used for classification are determined, classification models can be trained using the available EIS measurements. There are many machine learning algorithms that can be used to solve the classification problem. Logistic regression, an established and widely used classification method in medical/clinical data analysis [15] for disease diagnosis, was selected in this study to solve our problems of HG CIN detection and evaluating the prognostic value of EIS due to its simplicity and interpretability.

Logistic regression is concerned with direct modelling the posterior probability *p*(*C_1_|*x**)that an instance belongs to a particular class or group C1 (e.g. women likely to have HG CIN for problem of HG CIN detection, or women likely to develop HG-CIN within follow-up years for problem of evaluating prognostic value of EIS) given the observed feature vector ***x*** , In logistic regression, this posterior probability *p*(*C_1_|*x**) is modelled with the logistic function defined below [11]:


(4) $$P\left(C_{1}|x\right)=\frac{1}{1+e^{-a(x)}}$$

where a(*x*), in the basic form, is a linear function of x defined as.


(5) $$a(x)=\beta^{T}\left[\begin{array}{l} 1\\ x \end{array}\right]$$

and the regression coefficient vector 𝛽 (with up to 9 elements i.e. $\beta ={\left[\begin{array}{lllll} \beta_{0} & \beta_{1} & \beta_{2} & \cdots & \beta_{8} \end{array}\right]}^{T}$in this study) will be estimated from the training data. Classification using the above linear logistic regression model will result in a linear decision boundary (hyperplane $a(x)=\left[\begin{array}{ll} 1 & x \end{array}\right]\beta =0)$which does not have enough flexibility for classifying the data that is not linearly separable. However, it can easily be extended to obtain a non-linear decision boundary by using e.g. polynomial functions of the predictors. In general, a(*x*) can be expressed as:


(6) $$a(x)=\beta_{0}+\sum_{i=1}^{k}\beta_{i}\varphi_{i}(x)$$

where $\varphi_{i}(x)(i=1,\cdots ,k)$are some known (e.g. polynomial) functions of ***x***, In such a case, a(*x*) is still linear-in-the-parameters and can actually be viewed as the linear logistic regression model in terms of new features/or predictors $\varphi_{i}(x)(i=1,\cdots ,k).$

However, a major challenge for using the above model in this study is the determination of the model structure and evaluating the performance of the corresponding model with a class-imbalanced EIS data set of limited size. The problem is particularly severe in the data set used for the longitudinal study to evaluate the prognostic value of EIS, where the number of women who developed HG-CIN within three follow-up years in the whole population is very small (35 of 569). Hence simple partitioning of the data into two (i.e. training and test) sets for building and validating model may not work as this is likely to result in substantially different class distributions between the training and test sets and even no HG-CIN sample at all in some sets. To overcome this difficulty, *k*-fold cross validation with stratified random sampling (see e.g. [12]) was used to evaluate the classification performance so as to determine the optimal model structure to be used in the final model, this includes determining the degree of the polynomial to be used and the terms (i.e. 𝜑_*i*_(*x*)) to be included in the final model. For HG CIN detection, the size of available EIS data set is relatively large (1704), so 2-fold (training/testing) cross validation with stratified random sampling was applied to the data set, where one fold of size 1000 was used to train the classification model and another fold of size 704 was used to evaluate the classification performance of the trained model so as to determine the best model structure to be used. The two folds of data were constructed by first proportionately and randomly partitioning the original data in each class group into two subsamples, then merging a subsample from each class group to form a fold such that each fold contains roughly the same proportions of the two types of classes as in the original population. For the longitudinal study, 5-fold cross validation with stratified random sampling was used. Specifically, the original EIS data in each class group was randomly partitioned into 5 equal sized subsamples respectively. A fold was then constructed by merging a single subsample from each class group and this ensured that each fold contains roughly the same proportions of the two types of classes as in the original population (in this case, each fold will contain 7 women who developed HG-CIN) and the 5-fold cross validation procedure was then used to choose the best classification model to be used for evaluation of prognostic value of EIS for cervical cancer diagnosis.

As can be seen, the logistic regression model defined by equations (4) and (6) is computationally simple. The posterior probability *p*(*C_1_|*x**) is expressed as an explicit function of the features, hence has good interpretability. This allows us to get a better idea about the relationship between the increased risk of having or developing HG-CIN and the changes in cervix tissue structure.

### Informed consent

Informed consent has been obtained from all individuals included in this study.

### Ethical approval

The research related to human use has been complied with all relevant national regulations, institutional policies and in accordance with the tenets of the Helsinki Declaration, and has been approved by the authors’ institutional review board or equivalent committee.

## Results

Following the discussion in the last section, the area under the receiver operating characteristic (ROC) curve (abbreviated as AUC), a commonly used index for measuring the performance of classifier [16], together with the stratified *k*-fold cross validation procedure discussed previously, were used in this study for evaluating the classification performance of various logistic regression models so as to determine the final models to be used for HG CIN detection and evaluation of the prognostic value of the EIS respectively.

### Results for HG CIN detection

The EIS device ZedScan^TM^ has been developed as an adjunct diagnostic device to be used alongside colposcopy to provide an objective assessment of the cervical epithelial tissue in real time so that the colposcopist can take the ZedScan results into account when reaching their decision on patient management. With the current template matching method for HG CIN detection, the EIS device is programmed so that the threshold used for any given patient will depend upon the referral cytology result and also whether the colposcopist has identified the presence of HG CIN (i.e. colposcopic impression (CI), see [5]). In other words, the clinical information (i.e. referral cytology result and CI which can be viewed as two qualitative variables that taken values of either HG CIN or non-HG CIN) has been integrated into the template matching-based diagnostic decision making procedure when using ZedScan^TM^ in clinic practice. The new logistic regression classification-based method for HG CIN detection developed in this paper will be used in a similar way with the same setting. Hence in addition to the quantitative features/predictors defined in (3), the qualitative clinical information mentioned above also need to be incorporated into the logistic regression model. This can be done with two dummy variables (denoted as CI and Ref hereafter) that take on two numerical values (e.g. 1= HG CIN and 0=non-HG CIN) and the full expression of a(*x*) in the logistic regression model can then be re-written as:


(6) $$a(x)=\beta_{0}+\sum_{i=1}^{k}\beta_{i}\varphi_{i}(x)+\beta_{\text{CI}}\cdot \text{CI}+\beta_{\mathrm{Ref}}\cdot \mathrm{Ref}$$

where $\beta_{i}(i=1,\cdots ,k)$are the coefficients associated with the terms derived from EIS readings, coefficients $\beta_{\text{CI}}$and βRef determine the strength of influence of the corresponding clinical information on the possibility of patient being HG CIN which, together with $\beta_{i}(i=$0,1,⋯,k),will be learnt from the training data set.

Extensive studies have been carried out to evaluate the diagnostic performance of logistic regression models with different structures, i.e. the models constructed with different polynomial terms (up to degrees 3) of the selected features in x using the stratified 2-fold cross validation procedure with a 1000/704 training/testing split described previously. To reduce the uncertainty in the performance estimates, the procedure was repeated 10 times for each model and a different splitting of the dataset into 2 folds was implemented (via random permutation of data points in two groups respectively) for each repetition. The AUCs of ROC for the testing data sets of each repetition were summarized in Table 3 below, where three models with the best mean AUC values over 10 repetitions for polynomial degrees 1, 2, and 3 respectively are listed and the terms of the regression model are specified in the first row of the table.

**Table.3:** AUC values for testing sets from 10 repeated two-fold cross validation runs with three logistic regression models

Repetitions	$\begin{array}{l} {\bar{R}}_{0},\bar{\alpha },\Delta R_{0},\\ \text{ CI,Ref} \end{array}$	$\begin{array}{l} {\bar{R}}_{0}^{2},{\bar{\alpha }}^{2},\Delta R_{0}^{2},\\ \text{ CI,Ref} \end{array}$	$\begin{array}{l} {\bar{R}}_{0}^{3},{\bar{\alpha }}^{3},\Delta R_{0}^{3},\\ \text{ CI, Ref} \end{array}$
1	0.9127	0.9160	0.9165
2	0.9177	0.9178	0.9190
3	0.9013	0.9034	0.9045
4	0.9165	0.9210	0.9238
5	0.8830	0.8840	0.8858
6	0.9206	0.9222	0.9230
7	0.9164	0.9165	0.9172
8	0.9053	0.9061	0.9075
9	0.9122	0.9146	0.9155
10	0.9181	0.9215	0.9222
Mean AUC	0.9104	0.9123	0.9135

It can be seen that the model corresponding to the Column 4 of Table 3 has the largest mean AUC value among three models, so the final model structure is specified by the first row of Column 4 and a(*x*) in the final logistic regression model is then defined as:


(7) $$a(x)=\beta_{0}+\beta_{1}{\bar{R}}_{0}^{3}+\beta_{2}{\bar{\alpha }}^{3}+\beta_{3}\Delta R_{0}^{3}+\beta_{\text{CI}}\cdot \text{CI}+\beta_{\text{Ref}}\cdot \mathrm{Ref}$$

Once the final model structure is determined, all the coefficients in (7) can be estimated using the entire 1704 EIS data and the resulting estimated coefficients and the associated *p*-values are summarized in Table 4, As can be seen in Table 4, at the usual 5% significance level, all coefficients can be considered as statistically significant.

**Table.4:** Regression coefficient estimates and the associated *p*-values for the final logistic regression model

*β*	estimates	*p*-values
*β*o	-2.9619	3.9518 × 10^−32^
*β*1	−7.4684 × 10^−11^	0.0047
*β*2	3.3987	0.0090
*β*1	3.0025 × 10^−11^	0.0044
*β*CI	2.3621	3.9281 × 10^−47^
*β*Ref	2.2241	5.8068 × 10^−35^

**Fig.3:**
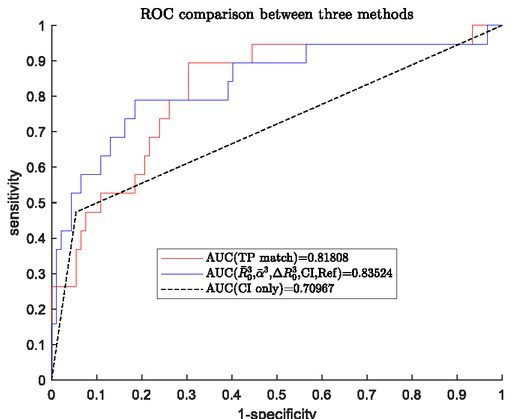
ROC comparison between new method, template match method and colposcopy only.

To validate the new method for HG CIN detection developed above and to compare the performance of the new method with that of the template match method currently used, the new method with the final model (7) and coefficients given in Table 4 was applied to a new set of EIS data from Royal Free Hospital in London. The size of this new data set was relatively small with severe class-imbalance (17.12% of HG CIN), N= 111 patients. The ROC curve from new method is shown in Figure 3, For comparison, the ROC curves from the template match method currently used, as well as colposcopy only are also displayed in Figure 3, where the blue line is the ROC from the new method with the logistic regression model specified by equation (7) and AUC=0.83524, the red line was the ROC from template match method with AUC=0.81808.

It can be seen that the new method can achieve similar performance as the template match method and both of them outperform colposcopy alone. Figure 3 shows a clear improvement in diagnostic performance when EIS is used (with either the new method developed in this paper or the current template match method) alongside colposcopy in comparison with colposcopy alone.

### Results for evaluation of prognostic value of EIS

The research on the evaluation of prognostic value of EIS carried out in this paper is the continuation of the study presented in [10]. All the women in the study had a negative outcome at their initial colposcopy and were then followed up for three years. The main objective of the research was to see if we were able to identify any increased risk of HG-CIN developing over the follow-up years based on the EIS readings taken at the initial colposcopy so as to evaluate the prognostic value of EIS readings.

The stratified 5-fold cross validation procedure discussed previously was applied to the data set taken from 569 women who had been followed up to three years so as to determine the final model to be used for evaluating the prognostic value of the EIS and the results were summarized in Table 5 and 6 below, where Columns 1 and 3 of these tables specify the feature combinations used for building the logistic regression models and columns 2 and 4 show the corresponding mean AUC values from 100 repeated 5-fold cross validation runs. A different partitioning of the dataset into 5 folds was implemented (via random permutation of data points in two groups respectively) for each run.

**Table.5:** Mean AUC values from 100 5-fold cross validation runs with linear logistic regression models

Feature combinations	Mean AUC	Feature combinations	Mean AUC
$\bar{\alpha },\Delta \alpha$	0.5870	${\bar{R}}_{0},\;\alpha ,\;\Delta \alpha$	0.5723
$\bar{\alpha },\Delta R_{\infty }$	0.5777	${\bar{f}}_{c},\alpha ,\Delta \alpha$	0.5716
${\bar{f}}_{c},\bar{\alpha }$	0.5745	$\bar{\alpha },\Delta R_{0}$	0.5715
$\bar{f_{c}},\;\Delta \alpha$	0.5744	$\bar{\alpha },\Delta f_{c}$	0.5686
${\bar{f}}_{c},\Delta R_{\infty },\Delta \alpha$	0.5736	${\bar{R}}_{0},\bar{\alpha }$	0.5678

**Table.6:** Mean AUC values from 100 5-fold cross validation runs with nonlinear logistic regression models

Feature combinations	Mean AUC	Feature combinations	Mean AUC
${\bar{\alpha }}^{2},\Delta \alpha^{2}$	0.6103	${\bar{f}}_{c},{\bar{\alpha }}^{2},\Delta \alpha^{2}$	0.5911
$\Delta \alpha ,{\bar{\alpha }}^{2}$	0.5992	${\bar{R}}_{0}^{2},\alpha^{2},\Delta \alpha^{2}$	0.5899
$\bar{\alpha },\Delta \alpha^{2}$	0.5989	$\Delta R_{\infty },{\bar{\alpha }}^{2},\Delta \alpha^{2}$	0.5895
${\bar{\alpha }}^{2},\bar{\alpha }\cdot \Delta \alpha ,\Delta \alpha^{2}$	0.5946	$\Delta R_{\infty }^{2},{\bar{\alpha }}^{2},\Delta \alpha^{2}$	0.5891
$\alpha ,\;\Delta \alpha ,\;\Delta \alpha^{2}$	0.5939	${\bar{\alpha }}^{2},\Delta f_{c},\Delta \alpha^{2}$	0.5885

Table 5 shows the ten linear combinations of features for building logistic regression models that have the largest mean AUC values among all possible linear combinations of 8 features defined in (3). As can be seen from Table 5, including more features in the linear logistic regression model does not necessarily improve the classification performance and the best linear logistic regression model (in terms of mean AUC value) is constructed with $\bar{\alpha }\text{ and }\Delta \alpha .$This is in agreement with the results obtained from the multivariate analysis of variance carried out previously.

**Fig.4:**
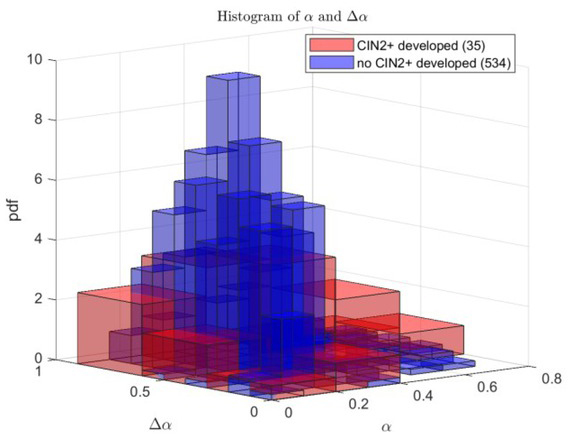
2-D histogram of α̅-Δα data points from two groups

Table 6 shows the ten nonlinear combinations of features for building the logistic regression models that have the largest mean AUC values among all possible nonlinear combinations of (up to the second order polynomial) 8 features. Similarly, it can be seen from Table 6, using more features/or polynomial terms in the logistic regression model does not necessarily improve the classification performance and the best nonlinear logistic regression model (in terms of mean AUC value) is constructed with the polynomial terms ${\bar{\alpha }}^{2}\text{ and }\Delta \alpha^{2},$hence it has an ellipse decision boundary. Figure 4 below is the 2-D histogram of the feature data $\bar{\alpha }\text{ and }\Delta \alpha .$It can be observed that the $\bar{\alpha }\text{-}\Delta \alpha$data points from women who did not develop HG-CIN within follow-up years tend to be concentrated in an area relatively close to the origin; whereas the data points from women who did develop HG-CIN within follow-up years tend to be distributed over the outskirts of this area away from the origin which means that those women tend to have large α̅ or/and Δαvalues.

Once the “winning” model structure was determined, we could then train this model with the whole date set to finalize our classification model and determine the optimal operating point (OOP). In this case, the “winning” model was constructed with the polynomial terms ${\bar{\alpha }}^{2}\text{ and }\Delta \alpha^{2},\text{so }a(x)$in the final logistic regression model for evaluation of prognostic value of EIS was defined as:


(8) $$a(x)=\beta_{0}+\beta_{1}{\bar{\alpha }}^{2}+\beta_{2}\Delta \alpha^{2}$$

**Fig.5:**
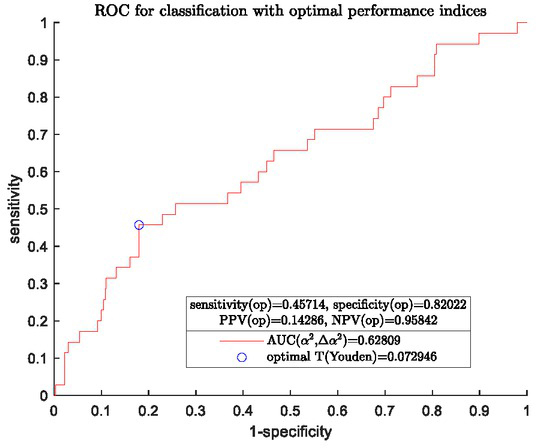
An ROC curve of final model for separating two groups with OOP and the associated performance indices

The OOP was chosen in this study such that Youden index [17] *J*=sensitivity+specificity-1 was maximized. This could readily be obtained from the ROC curve of the final model and the results are shown in Figure 5.

In the previous study reported in [10], two single features derived directly from mean spectra of individual women, i.e. the impedance at 152Hz and the slope of the EIS spectra between frequencies 1.22 and 2.44kHz (used as a proxy for α),were respectively used to build a classifier for separating the two groups. The classification performance for the given data set of these two classifiers were compared with that of the new logistic regression classifier determined by equation (8) and the results were summarized in Table 7.

**Table.7:** Classification performance comparison between the new classifier developed and the previous classifiers

Classifier	AUC	Sensitivity	Specificity
Logistic regression $\left({\bar{\alpha }}^{2},\Delta \alpha^{2}\right)$	0.628	45.714%	82.022%
Impedance at 152Hz	0.621	38.7%	83.4%
Slope (between 1.22	0.596	45.2%	70.1%
and 2.44kHz) as α			

In Table 7, the sensitivity and specificity were calculated at the OOP determined from ROC curves of the corresponding classifiers. It can be seen from Table 7, overall, the performances from the logistic regression classifier developed in this paper and the previous classifiers are comparable, but the new classifier can achieve relatively balanced sensitivity and specificity. More importantly, with

the new classifier, the possibility of women who could develop HG CIN within follow-up years is expressed as an explicit function of the features $\bar{\alpha }\text{ and }\Delta \alpha$via equation (8), this will allow us to associate the risk of developing HG CIN within follow-up years with the tissue structure change caused by the evolution of neoplasia.

## Discussion

The two objectives of the research in this paper were: 1.) to develop a template-free EIS data analysis method for disease detection to enable the EIS–based techniques to be used for new areas of medical diagnosis where the template spectra are not available; 2.) In addition to being template-free, the developed method should also provide information on how the changes in cervical tissue structure/property due to disease could be reflected in the changes of the observed EIS spectra, this would ultimately help us to better-understand the mechanism that underpins the EIS-based disease detection. To achieve the first objective, a data-driven approach in combination with machine learning, or more specifically classification, techniques were employed in this study to develop the new EIS data analysis method. To achieve the second objective, a Cole model-based spectrum curve fitting approach was developed to extract features from EIS readings for classification and a logistic regression technique was used to build interpretable classification models for HG CIN detection and evaluation of prognostic value of EIS. This enabled us to associate the probability of HG CIN being present, or developing HG CIN later, with the change in tissue structure due to disease via Cole parameter estimates.

Two logistic regression models, as specified by equations (7) and (8), were developed using real service EIS data from the Jessop Wing Colposcopy clinic in Sheffield, one for HG CIN detection and another one for evaluation of prognostic value of EIS. With the logistic regression model specified by (7), the probability of HG CIN being present given the EIS readings is expressed as an explicit function of the features ${\bar{R}}_{0},\bar{\alpha }\text{ and }\Delta R_{0}.$This actually establishes some histopathologally interpretable links between the probability of detecting HG CIN and the changes in tissue structures due to disease. For example, CIN leads to the increase in the extracellular space which, in turn, results in the decrease in ${\bar{R}}_{0}$(as the inverse of extracellular volume determines R0). As 𝛽_1_ (regression coefficient associated with ${\bar{R}}_{0}^{3})$in equation (7) has a negative sign, this finally increases the probability of detecting HG CIN. Hence, this classification model provides us with useful information to understand how the changes in the tissue structure and properties could increase the risk of HG CIN being present.

The new method had been validated using a set of real EIS data from Royal Free Hospital in London and the classification performance was comparable to that of the template match method currently used with the EIS device ZedScan^TM^ , This demonstrates the usefulness of the methodology and the associated core algorithms developed. As the new method is purely data driven, it can readily be extend to other areas of medical diagnosis where the template spectra are not available, e.g. oral cancer diagnosis [18]. In addition, it can be observed from Figure 3 that, though the new method and the template match method offer similar classification performance overall, there are some subtle differences. The new method tends to have a slightly higher specificity, whereas template match method tends to have a slightly higher sensitivity. Based on this observation, it might be possible to improve the overall diagnostic performance by combining or integrating two methods together. This can be done by taking the score from the template match method as an extra feature to build and train a new logistic regression model for classification. This is another research topic that is being carried out by the authors, but it is out of the scope of this paper.

The logistic regression model specified by equation (8) had been developed with the data set for evaluation of the prognostic value of EIS. It shows that the increased risk of developing HG CIN within follow-up years essentially depends on the handcrafted features $\bar{\alpha }\text{ and }\Delta \alpha ,$which are determined by the inhomogeneity of the cells within the tissue (i.e. the diversity of cell size and structure) and the spatial inhomogeneity of the tissue around the cervix. This is very reasonable as these are the features or properties associated with an evolving cervical neoplasia. Equation (8) actually verifies the speculation postulated in [10] that the increased risk of developing HG-CIN is associated with the increased diversity of cellular structures or inhomogeneity. Comparing models (7) and (8), we can see that once the neoplasia becomes more severe and/or has transferred into HG CIN, the extracellular volume that determines ${\bar{R}}_{0}$becomes another important property to differentiate between normal and cancerous tissues.

A weakness in this study is that the classification performance with the model specified by equation (8) was not so great as can be seen in Figure 5 in comparison with that of Figure 3 for HG CIN detection. This is, in some extent, expected and in agreement with the previous result from multivariate analysis of variance because it is more difficult to identify early signs caused by the incipient change in tissue properties as neoplasia evolves at its early stage than to detect signs that caused by a severe neoplasia or HG CIN. Another contributing factor is the limited data set available, in particular, the small portion of women who developed HG CIN within the follow-up years in the study population. It also needs to be pointed out that the result presented in Figure 5 is based on EIS only, it may be possible to further improve performance by incorporating some clinical information into the classification model as we did for HG CIN detection. Nevertheless, the results do reach statistical significance, hence EIS does contain prognostic information on evolving cervical neoplasia, which provides important information that should be useful for the development of a practical patient management scheme following a negative colposcopy.

To sum up, the two main novelties of the methodology developed in this paper are: 1.) to introduce a Cole model-based spectrum curve fitting approach to extract features from EIS readings for classification. This allows the increased risk of HG CIN being present or developing HG CIN to be associated with the changes in tissue structure due to disease and helps us to understand the underpinning mechanism of EIS-based disease detection. 2.) to introduce the maximum differences of the Cole model parameter estimates over all reading sites around the cervix as features, in addition to the Cole parameter estimates of the mean spectra. These maximum differences can be viewed as a measure for the spatial inhomogeneity of tissue around cervix and allow the small or incipient lesions to be detected. The signs due to these small or incipient lesions could be smoothed out by averaging or covered by diversity of conditions between individual patients, hence may be difficult to be detected using features derived from the mean spectra alone.

The single dispersion Cole equation had been used in this study for feature extraction. Because it appeared to be the case that EIS spectra taking from cervical tissue were dominated by a single dispersion. However, it needs to be pointed out that in other applications such as oral cancer diagnosis, there may be more than one identifiable dispersion. In such a case, it may be necessary to use a multiple dispersion Cole equation for feature extraction.
